# Supplementary material for: Establishment of a screening platform based on human coronavirus OC43 for the identification of microbial natural products with antiviral activity
Source: Microbiol Spectr. 2023 Nov 27;12(1):e01679-23. doi: 10.1128/spectrum.01679-23 (PMC10783114; doi:10.1128/spectrum.01679-23)
Supplement: Supplemental material — Fig. S1 and S2. [file spectrum.01679-23-s0001.pdf]

## SUPPLEMENTAL FIGURES

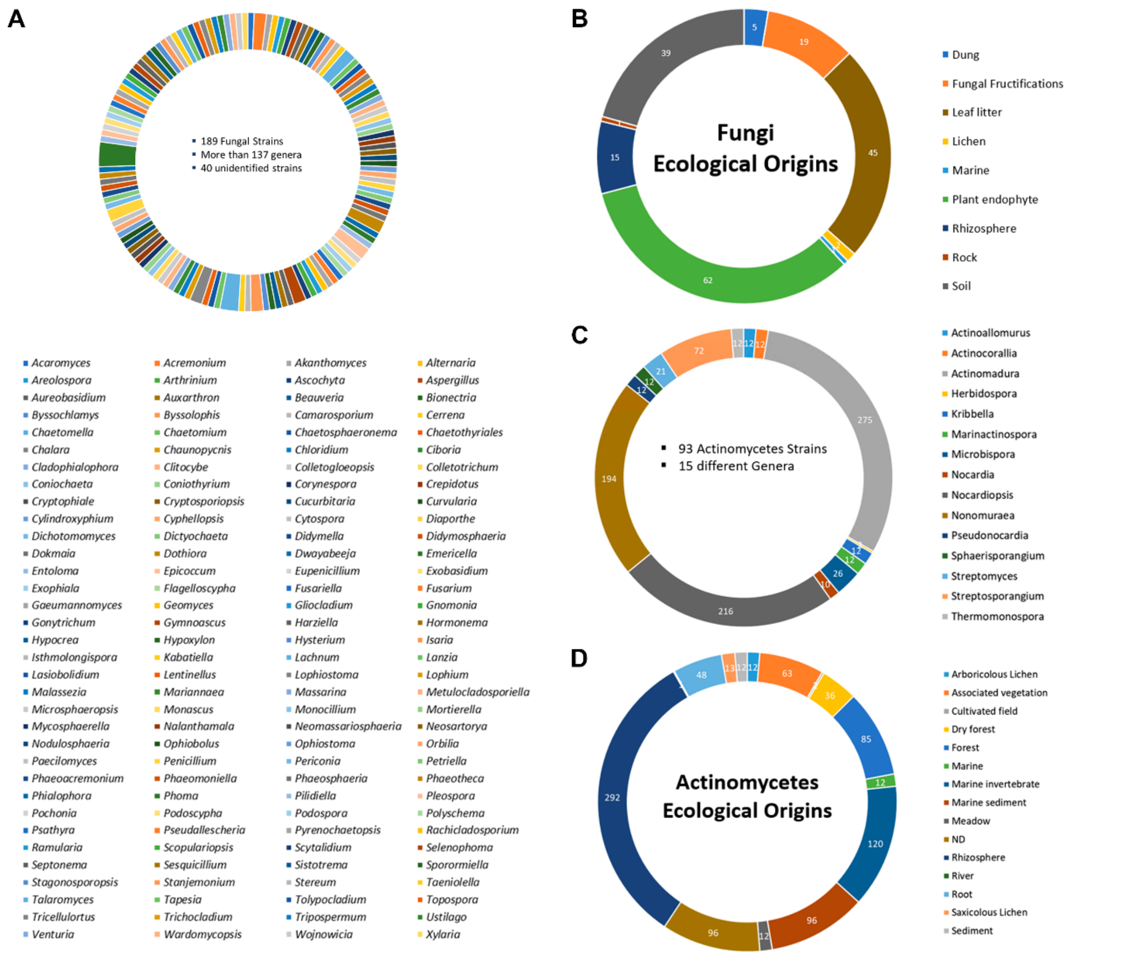


Supplemental Figure 1. Selected 1280 microbial extracts from MEDINA's Natural Product library. A. Genera of fungal extracts. B. Ecological origins of the 189 fungal strains. C. Genera of actinomycetes extracts. D. Ecological origin of the 93 actinomycetes strains.

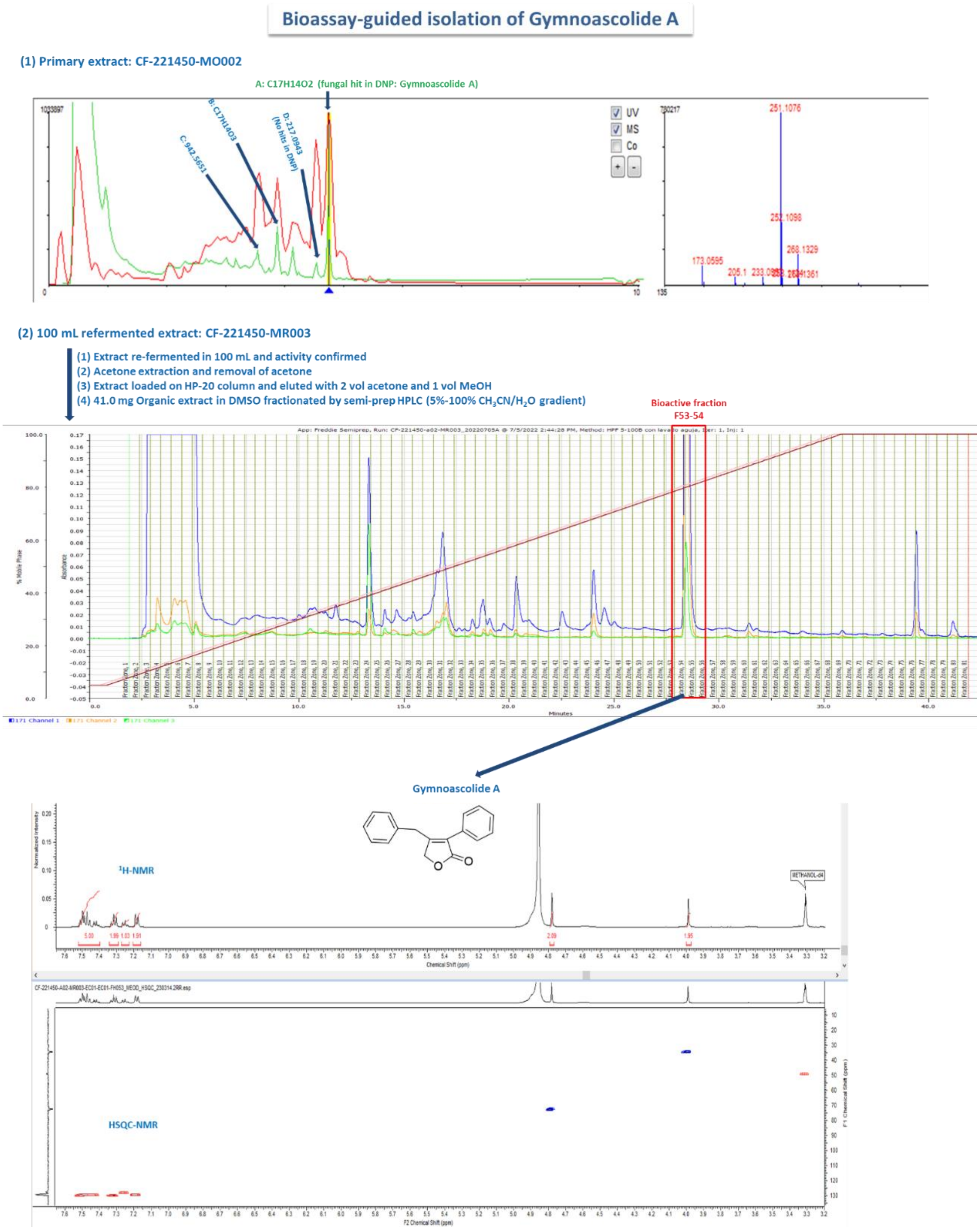


Supplemental Figure 2. Bioassay-guided isolation of gymnoascolide A from 100 mL re-fermentation of CF-221450-MR003 extract (ID extract 3). (1) Shows the LC-HRMS profile of the primary active extract from which gymnoascolide A (highlighted in green and its mass spectrum shown on the right) was initially identified as a main component. (2) Shows the stepwise bioassay-guided isolation process through which the 100 mL

re-fermented extract was taken, the eventual isolation of gymnoascolide A from HPLC fraction F53-54, and the $^{1}H$ and HSQC NMR confirmation of the compound identity.
